# Radiomic and deep learning characterization of breast parenchyma on full field digital mammograms and specimen radiographs: a pilot study of a potential cancer field effect

**DOI:** 10.1117/1.JMI.10.4.044501

**Published:** 2023-07-08

**Authors:** Natalie Baughan, Hui Li, Li Lan, Matthew Embury, Isaiah Yim, Gary J. Whitman, Randa El-Zein, Isabelle Bedrosian, Maryellen L. Giger

**Affiliations:** a The University of Chicago, Department of Radiology, Chicago, Illinois, United States; b The University of Texas MD Anderson Cancer Center, Department of Breast Surgical Oncology, Houston, Texas, United States; c The University of Texas MD Anderson Cancer Center, Department of Breast Imaging, Houston, Texas, United States; d The Houston Methodist Research Institute, Houston, Texas, United States

**Keywords:** breast cancer risk assessment, breast parenchymal patterns, radiomics, deep learning, image analysis

## Abstract

**Purpose:**

In women with biopsy-proven breast cancer, histologically normal areas of the parenchyma have shown molecular similarity to the tumor, supporting a potential cancer field effect. The purpose of this work was to investigate relationships of human-engineered radiomic and deep learning features between regions across the breast in mammographic parenchymal patterns and specimen radiographs.

**Approach:**

This study included mammograms from 74 patients with at least 1 identified malignant tumor, of whom 32 also possessed intraoperative radiographs of mastectomy specimens. Mammograms were acquired with a Hologic system and specimen radiographs were acquired with a Fujifilm imaging system. All images were retrospectively collected under an Institutional Review Board-approved protocol. Regions of interest (ROI) of 128×128  pixels were selected from three regions: within the identified tumor, near to the tumor, and far from the tumor. Radiographic texture analysis was used to extract 45 radiomic features and transfer learning was used to extract 20 deep learning features in each region. Kendall’s Tau-b and Pearson correlation tests were performed to assess relationships between features in each region.

**Results:**

Statistically significant correlations in select subgroups of features with tumor, near to the tumor, and far from the tumor ROI regions were identified in both mammograms and specimen radiographs. Intensity-based features were found to show significant correlations with ROI regions across both modalities.

**Conclusions:**

Results support our hypothesis of a potential cancer field effect, accessible radiographically, across tumor and non-tumor regions, thus indicating the potential for computerized analysis of mammographic parenchymal patterns to predict breast cancer risk.

## 1 Introduction

Breast cancer is the most commonly diagnosed cancer for women in the United States, and is estimated to be diagnosed in approximately one in eight women in their lifetime.^1^ The Society of Breast Imaging and the American College of Radiology recommend annual mammography screening beginning at age 40 for women at average risk, and magnetic resonance imaging (MRI) or ultrasound screening as an adjunct to mammography for women classified to be at a higher risk.^2^ Screening mammography helps to reduce breast cancer-related mortality by enabling detection at earlier stages, when treatment is generally more effective and less invasive.^2^^,^^3^

Variations in screening frequency and age at mammography initiation are present in different recommendations from national and international bodies as a result of the balancing of the benefits and harms of additional screening in the general population.^3^^,^^4^ Current recommendations in the United States stratify average- from high-risk women primarily on the factors of family or personal history of breast cancer, gene mutation status, and history of chest irradiation. However, additional risk factors from mammography may provide information to help create a more personalized method of risk stratification, without the need for additional imaging or testing. Breast density is an example of one such factor that can be assessed from screening mammography and that is associated with an increased risk of breast cancer. However, breast density alone provides marginal improvement to screening sensitivity, as over 40% of screening-age women have been found to have heterogeneously or extremely dense breasts on mammograms, while the 5-year absolute risk of breast cancer is 1% to 2.5% for all screening age groups.^4^^,^^5^ As a result, mammographic density alone has not been found to have high enough sensitivity in prediction of future cancer to be used in most accepted risk stratification schemes.^2^^,^^4^

Previous studies have shown that artificial intelligence (AI) tools including human-engineered radiomic features and deep learning features extracted from mammograms have additive value to current breast cancer risk assessment metrics, including breast density.^6^^,^^7^ Radiomics and deep learning features offer methods to quantify a wide variety of parenchymal texture characteristics that are not based on radiologists’ judgment. However, most studies focus on the tumor region at the time of diagnosis, potentially overestimating the classification ability of such methods for a risk stratification application.

In women with biopsy-proven breast cancer, histologically normal areas of the parenchyma within the ipsilateral (and the contralateral) breast have shown molecular similarity to the tumor, supporting a potential cancer field effect. It is hypothesized that such an effect may be a precursor of malignancy or impact tumor recurrence.^8^ A field cancerization that is identifiable via mammography, and confirmation of the distance to which this cancerization extends into the normal adjacent tissue for various subtypes of breast cancer has yet to be confirmed in the literature. Identification of a cancer field effect in mammography has the potential to provide a novel approach to stratification of breast cancer risk in the general population by augmenting current risk assessment models. Radiomic texture analysis and deep learning are particularly well suited to identify and characterize potential signatures of a field effect in mammography due to their ability to quantify a multitude of image characteristics. A first step in this analysis is to investigate how these quantitative features may change with increasing distance from the tumor.

In this work, we used human-engineered radiomic features and deep learning features to identify and characterize texture signatures of a field effect in mammograms of women with biopsy proven breast cancer. A change in average feature values between breast parenchymal regions may be indicative of an image-based cancer field or cancer associated characteristics. Feature relationships as a function of the distance from the identified tumor could potentially aid in defining a cancer risk model. To the best of their knowledge, the authors believe this is the first study to investigate a field effect in mammography and the first to evaluate radiomic features of specimen radiographs and their relation to mammographic features.

## 2 Methods

### 2.1 Dataset

The dataset consisted of 103 retrospectively collected patients with at least 1 identified malignant tumor. Inclusion in the initial cohort was specified by patients who were diagnosed with breast cancer and had undergone mastectomy for treatment of their breast cancer at MD Anderson Cancer Center between 2010 and 2017. Known germline mutation carriers were excluded. Preoperative mammograms and intraoperative radiographs of the mastectomy specimens were retrieved under Health Insurance Portability and Accountability Act-compliant Institutional Review Board protocols. Patients with tumors occult on the craniocaudal (CC) view (n=18), no preoperative mammograms available (n=8), for presentation preoperative mammograms not available (n=2), and a breast region too small to fit a region of interest (ROI) (n=1) were excluded. The remaining 74 patients were used in the analysis. In addition, a subset of 32 patients had also undergone intraoperative radiographic imaging of the mastectomy specimen. In a conventional clinical setting, specimen radiographs are used to verify removal of the targeted abnormality and to evaluate the margins of the resection. Although evaluating specimen radiographs is not typical clinical practice for risk assessment, radiomic features of the tissue *in*- and *ex-vivo* may allow for a deeper understanding of the relationships of tissue texture for a potential cancer field effect. Mammograms were acquired with a Hologic (Marlborough, Massachusetts, United States) Lorad Selenia system (12-bit quantization, 70-micron pixels), and specimen radiographs were acquired with a Fujifilm (Lexington, Massachusetts, USA) imaging system (12-bit quantization, 50-micron pixels). Images were not pre-processed by the authors and all mammograms were the clinical “for presentation” images of diagnostic quality.

**Fig. 1:**
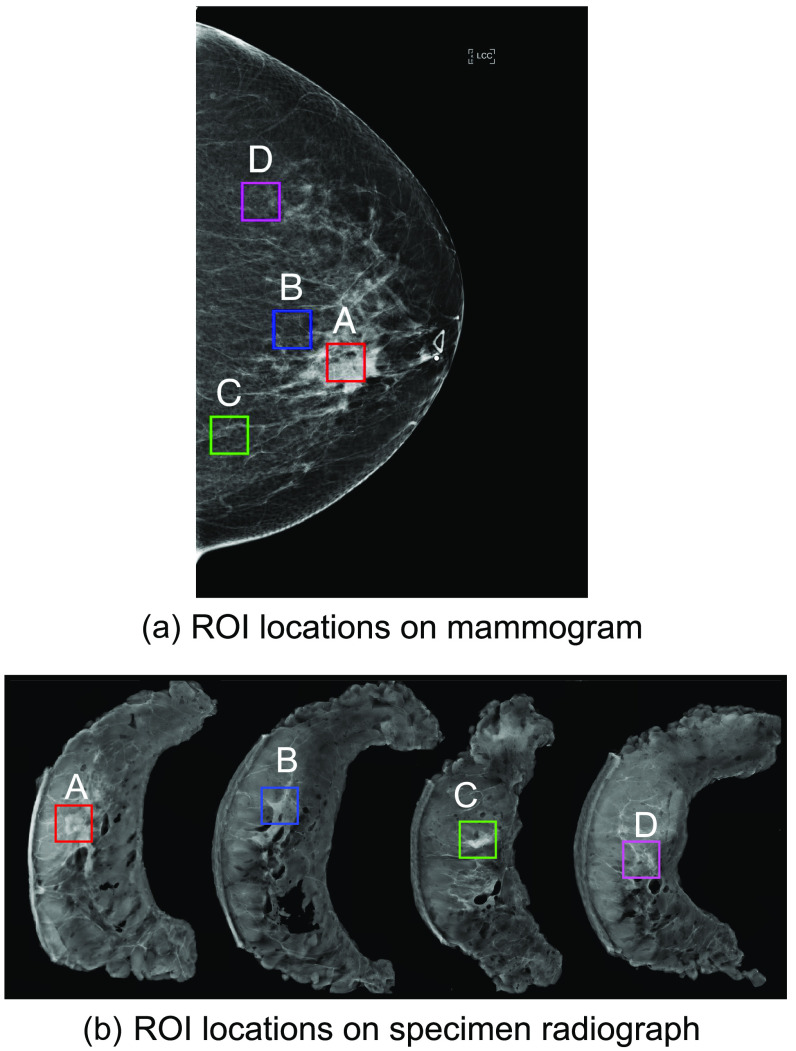
Example ROI locations for (A) tumor, (B) near to tumor, and (C and D) far from tumor regions on (a) CC-view mammogram and (b) the corresponding specimen radiograph for one selected patient. The specimen radiograph shows four serial sections of breast tissue from the same breast shown in panel (a).

The goal of this work is characterization of a potential field effect in women with confirmed breast cancer on mammograms and specimen radiographs. To accomplish this goal, ROIs of 128×128  pixels were selected from four regions on the CC mammogram projection: within the tumor (A), near to the tumor (B), and far from the tumor (C and D), as shown in Fig. 1(a). Size and location of the tumors were visually assessed with the assistance of a research specialist with over 15 years of experience in mammography. Near and far ROIs were placed by a naïve user with guidance of the tumor ROI location (to avoid overlap) and training to avoid major calcifications, markers, scars, and fatty tissue near the skin and the chest wall. If this was unachievable for a given patient, the ROI was not selected. Manual ROI placement enables certainty that ROIs will not include these objects that may impact quantitative feature values, but does not require a complex automation scheme for random placement. The “naïve user” was new to the field of image quantification and mammography and can be assumed to not have background knowledge necessary to bias ROI location given the tissue appearance. Near ROIs were placed in the closest “normal parenchyma” region to the tumor and far ROIs were placed as far from the tumor on the tumor side breast as could fit in the parenchymal tissue avoiding fatty tissue along the chest wall. Exact distance measurements were not specified as it is challenging to estimate given unknown compression pressure and three-dimensional distance to the tumor in a projection image. As such, the distribution of ROI locations used in this study is expected to represent average region characteristics, not characteristics of an exact location. For each paired mammogram and specimen radiograph analysis, corresponding 128×128  pixel ROIs were selected by a breast surgical oncologist with over 20 years of experience in the field from three regions across the serially sectioned specimen radiographs, as noted in Fig. 1(b): with in the identified tumor (A), near to the tumor (B), and far from the tumor (C and D). For specimen radiograph ROIs, distances of near and far ROI locations were designated as less than 2 cm from the tumor and greater than 2 cm from the tumor, respectively.

### 2.2 Feature Extraction

An in-house AI workstation was used to automatically extract 45 radiomic texture analysis features describing tissue contrast/intensity and structure in each breast region. Feature descriptions are listed in the Appendix Table 1 and formulas can be found in the literature.^9^^–^^13^ For deep learning-based features, a transfer learning approach was used. A VGG19 convolutional neural network architecture was first pre-trained on ImageNet.^14^ The generically trained network was then used with the mammogram and specimen radiograph ROIs as the input, and 1472 generic deep learning features were extracted from each of the 5 max pooling layers, similar to the approach described by Antropova et al.^15^ To select only features that were relevant to each data set, deep learning features with zero variance or features in which >50% of the values were zero were removed. To further reduce the number of features, principal component analysis was utilized to reduce dimensionality of the remaining features.^16^ The first 20 principal components (86.53% of the total variance for mammograms, and 89.67% of the total variance for specimen radiographs) were then used as pseudo-features, i.e., principal components serving as characteristic features, for each region.

### 2.3 Statistical Analysis

To assess correlation of features between ROI regions within a given image type, the Kendall’s Tau-b correlation test was used.^17^ This test allows for quantification of correlation between a categorical independent variable (ROI region) and a numerical dependent variable (feature values); thus, it was selected for evaluating correlations on mammograms and specimen radiographs separately. Kendall’s Tau-b is a nonparametric measure of the strength and direction of the association between two variables and is considered an alternative to the Spearman rank order correlation coefficient for data with many ties in each group.^17^^,^^18^

To evaluate correlations in features between mammograms and specimen radiographs, the Pearson correlation test was used.^18^ Pearson’s Rho is a commonly used measure of linear correlation between two variables. This test allows for quantification of correlation between two numerical variables, which is why it was selected to evaluate feature correlation between both modalities. For calculation of Pearson’s Rho, only matched pairs of patients and corresponding ROI regions with both mammograms and specimen radiographs were used (n=32 patients, 118 ROIs).

Both the metrics of Kendall’s Tau-b and Pearson’s Rho are bounded between −1 and 1, with values of zero indicating no correlation and one indicating the strongest correlation, with the sign indicating the direction of the relationship.

All hypothesis tests were adjusted for multiple comparisons using the Benjamini-Hochberg correction.^19^ This procedure controls for the false discovery rate (FDR), the proportion of significant results that are actually false positives. The Benjamini-Hochberg correction is recommended when the number of comparisons is large and is commonly used in exploratory procedures, such as identifying differentially expressed genes.^19^^,^^20^ In this correction, to be considered significant, the p-value must be less than the rank of said p-value (the smallest p-value would have a rank of 1, and the greatest p-value would have a rank of the total number of comparisons) divided by the total number of comparisons, multiplied by the selected FDR. Since this was completed for each set of tests, 45 was the total number of comparisons for radiomic features and 20 was the total number of comparisons for deep learning features. An FDR of 5% was selected to keep the number of potential false discoveries low, whereas FDRs of 10% to 25% are commonly used in genomic studies.^20^

## 3 Results

**Fig. 2:**
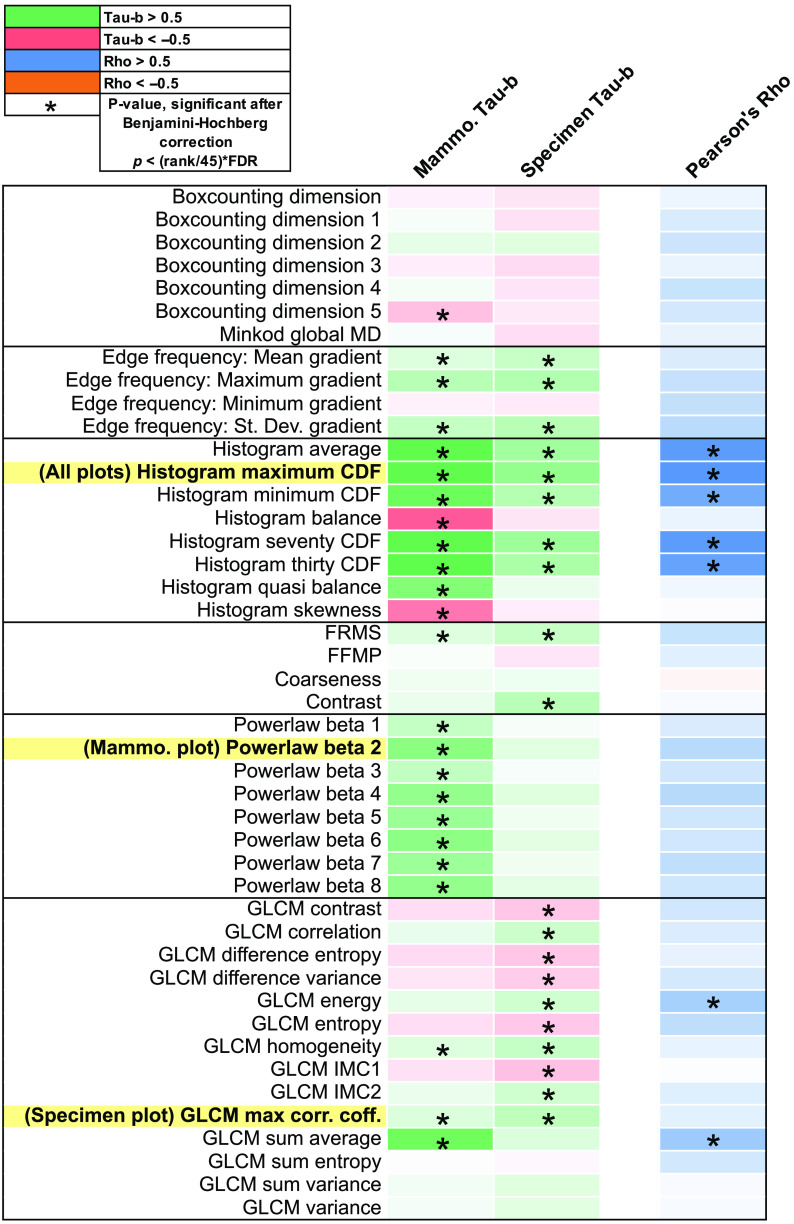
Table of Kendall’s Tau-b and Pearson correlation test results for radiomic features on mammography and specimen radiography. Kendall’s Tau-b was used to evaluate relationships between mammogram ROI regions and specimen radiograph ROI regions separately, and the Pearson’s correlation test was used to evaluate correlations between mammogram and specimen features. Feature names highlighted in yellow were selected to be plotted in Fig. 3. The color of each cell represents the direction and strength of each correlation as noted in the legend. The asterisks denote correlations considered significant after Benjamini-Hochberg correction with a 5% FDR.

Results of the Kendall’s Tau-b and Pearson correlation tests for all calculated radiomic features are shown in Fig. 2. Features were grouped into categories representing similar underlying characteristics. Color of cells for a given comparison represents the magnitude of the test statistic. For Kendall’s Tau-b, cells that are a more saturated green represent stronger positive correlations and cells that are a more saturated red represent stronger negative correlations. Similarly, for Pearson correlation, cells that are a more saturated blue represent stronger positive correlations and cells that are a more saturated orange represent stronger negative correlation. All color scales reached a maximum color saturation at a value of +/−0.5 and are shown in white for test statistics equal to zero. Asterisks in each cell represent correlations considered significant after Benjamini-Hochberg correction using a 5% FDR. Results of this test emphasize changes in the absolute values of features across the ROI regions.

For radiomic feature analysis, Kendall’s Tau-b test results indicated a majority of statistically significant correlations between the tumor, near, and far regions in mammograms for intensity-based histogram features, edge frequency features, and Fourier-based powerlaw beta features. In the specimen radiographs, results indicated a majority of statistically significant correlations between intensity-based histogram features, edge frequency features, and gray-level co-occurrence matrix (GLCM) features. Pearson correlation results identified a majority of statistically significant correlations in intensity-based histogram features between mammograms and specimen radiographs, presenting a strong relationship across the tumor, near, and far regions in both modalities. This result seems reasonable, given that tumors have been found to be more dense and coarser in texture than parenchymal tissue, while indicating strong correlations across tumor and non-tumor tissue.^7^^,^^16^^,^^17^

**Fig. 3:**
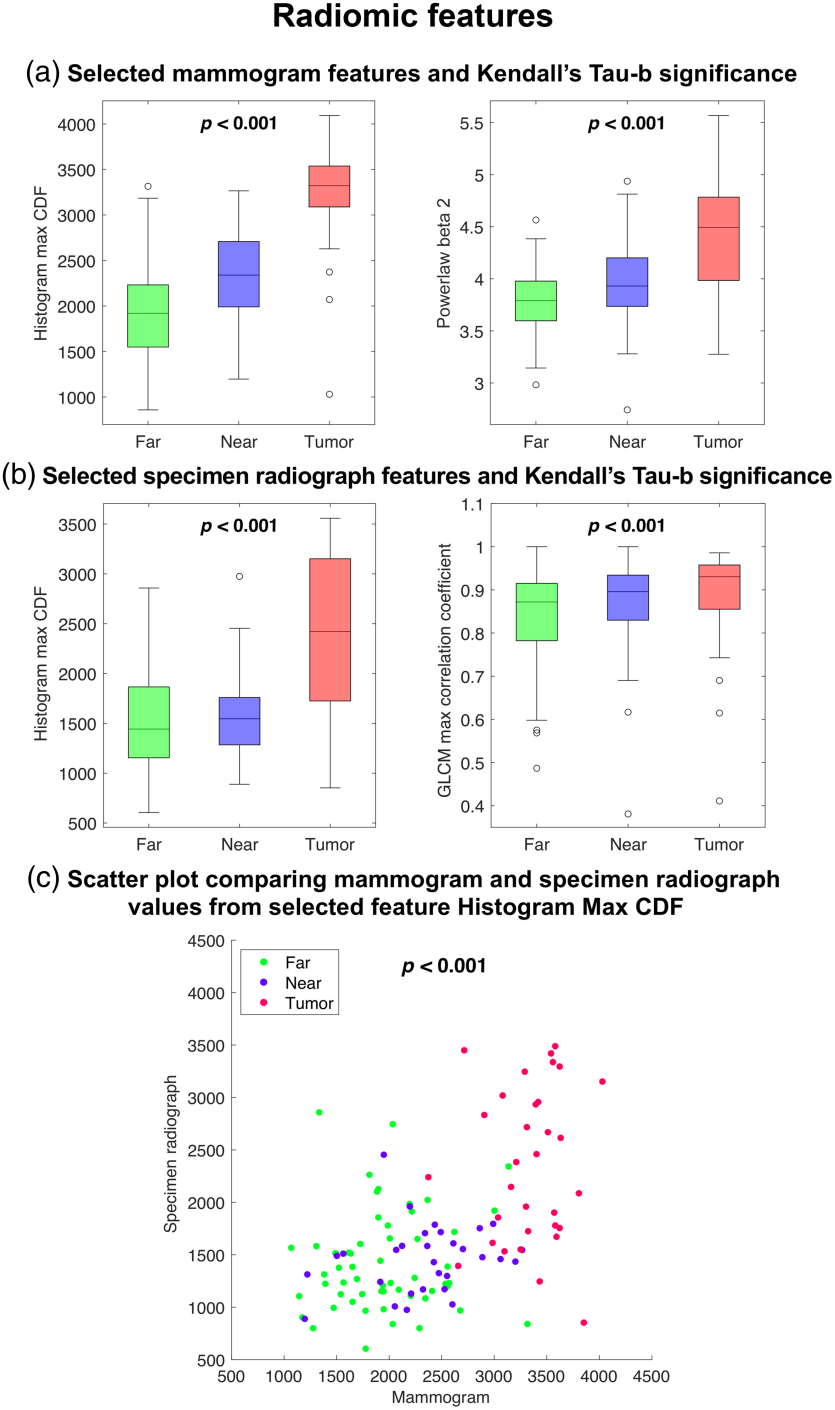
Boxplots of selected texture radiomic features in mammograms (a) and specimen radiograph texture features (b). Scatterplot of intensity-based histogram feature Max CDF, which had the strongest correlation between mammograms and specimen radiographs (c).

Highlighted features in Fig. 2 were selected as examples of the significant correlations from the radiomic feature analysis to be plotted in Fig. 3. Features were selected as follows: (1) histogram Max CDF – the strongest correlation for all mammogram and all specimen Kendall’s Tau-b and Pearson tests, (2) Powerlaw beta 2 – the strongest correlation in a subgroup of features where only the mammogram Kendall’s Tau-b test indicated statistical significance in a majority of the features, and (3) GLCM Max Correlation Coefficient – the strongest correlation in a subgroup of features where only the specimen radiograph Kendall’s Tau-b test indicated statistical significance in a majority of the features. Correlations indicated that radiomic features from ROIs closer to the tumor tended to show more similarity to the tumor than features from distant ROIs and there were statistically significant relationships of these features across the parenchymal field in *in-* and *ex-vivo* imaging.

**Fig. 4:**
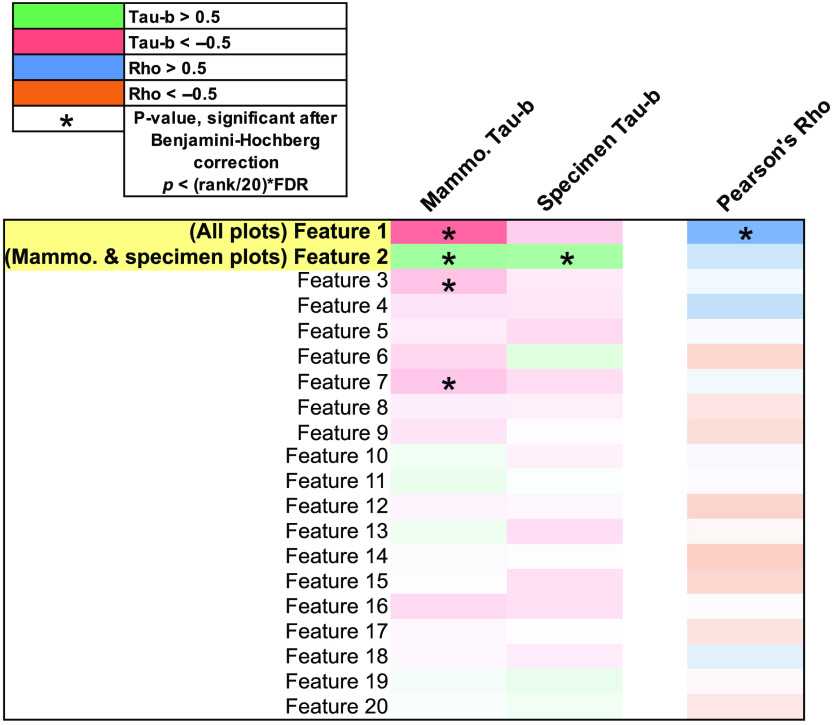
Table of Kendall’s Tau-b and Pearson correlation test results for deep learning features on mammography and specimen radiography. Kendall’s Tau-b was used to evaluate relationships between mammogram ROI regions and specimen radiograph ROI regions separately, and the Pearson’s correlation test was used to evaluate correlations between mammogram and specimen features. Feature names highlighted in yellow were selected to be plotted in Fig. 5. The color of each cell represents the direction and strength of each correlation as noted in the legend. The asterisks denote correlations considered significant after Benjamini-Hochberg correction with a 5% FDR.

Results of the Kendall’s Tau-b and Pearson correlation tests for all calculated deep learning features are shown in Fig. 4. Since the deep learning features do not carry easily definable categories and intuitive meanings as the radiomic features, it is important to note that the deep learning features represent principal components, and thus are listed in order of decreasing variance. The color scales and markers used to indicate correlation strength, direction, and significance are the same as described for radiomic features in Fig. 2.

For the deep learning feature Kendall’s Tau-b test in mammograms, correlations between the tumor, near, and far regions for the first three features and feature 7 were found to be statistically significant. In specimen radiographs, results indicated a statistically significant correlation in only feature 2. Pearson correlation results showed a statistically significant correlation in feature 1 between mammograms and specimen radiographs. These results seem reasonable, given that the first principal components/features will describe the majority of the variance in the dataset and the fundamental characteristics of the images.^16^

**Fig. 5:**
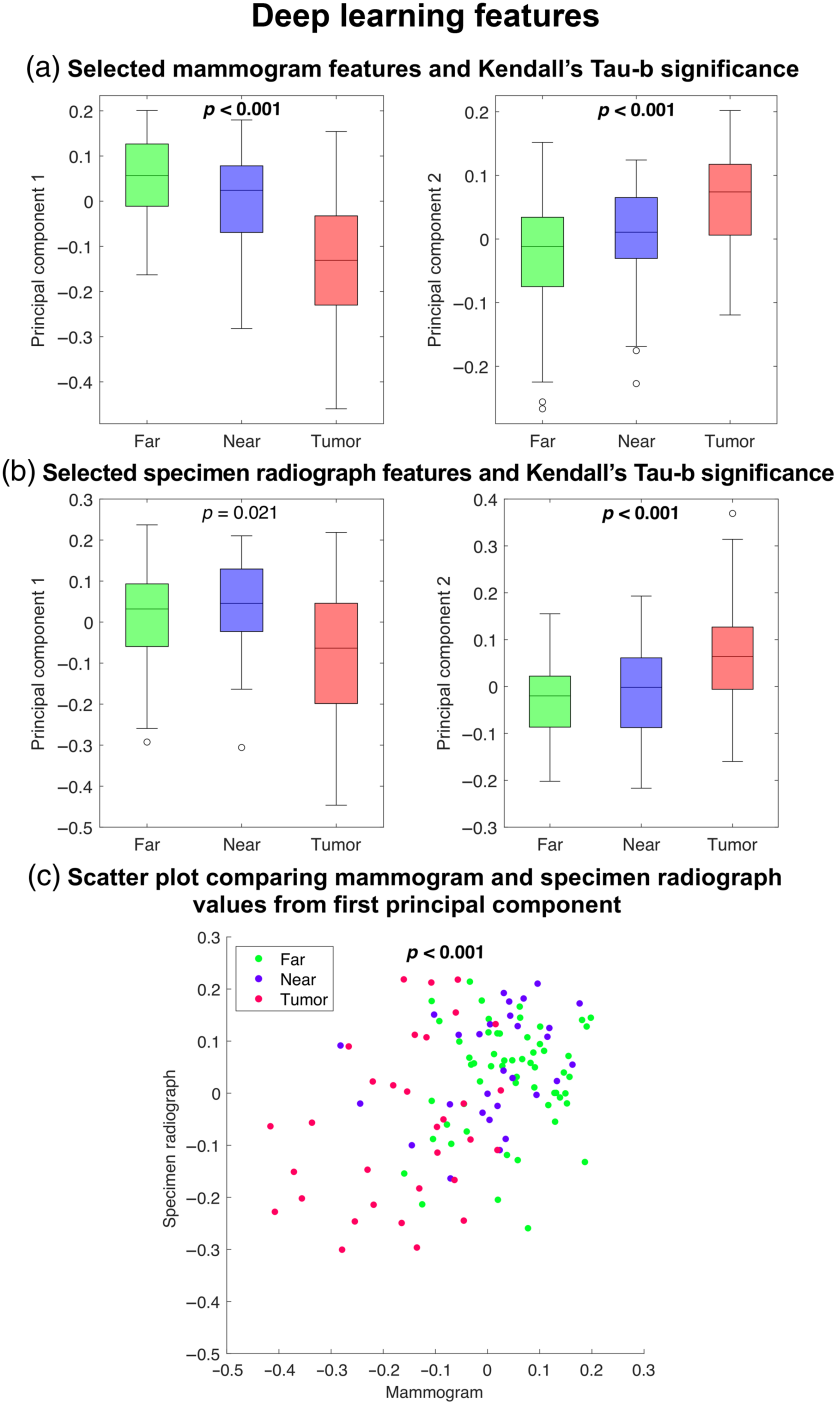
(a) Boxplots of selected deep learning features in mammograms and (b) specimen radiograph texture features. (c) Scatterplot of the first principal component, taken to be pseudo-feature 1, which had the strongest correlation between mammograms and specimen radiographs.

Highlighted features in Fig. 4 were plotted in Fig. 5 to demonstrate the correlations in the first two principal components from the deep learning feature analysis. Supporting the result found with radiomic features, correlations indicated that deep learning features from ROIs closer to the tumor tended to show more similarity to the tumor than features from distant ROIs in *in*- and *ex-vivo* imaging.

## 4 Discussion

Results of this study exemplify key relationships of the parenchymal field in women with cancer. Understanding these features and relationships will provide important information in understanding a potential mammography-based cancer field effect. Correlation results from radiomic features and deep learning principal component features showed preliminary evidence of a relationship between feature values and ROI location with increasing distance from the tumor.

For radiomic features calculated within the mammogram, statistically significant correlations were primarily identified in histogram or intensity-based, edge frequency, and Fourier-based features using Kendall’s Tau-b. Similarly, for deep learning features calculated within the mammogram, the first three and seventh principal components indicated statistically significant correlations. Although the underlying characteristics of principal component features cannot be understood in the same way as radiomic features, it can be established that the first principal components will represent the foundational characteristics of the object.^16^ Given this, it could be reasonable to infer that the first principal components may also quantify how light or dark the pixels from a given ROI are, describing the tissue intensity as well. This relationship across the mammographic field in intensity may be related to underlying density of breast tissue in a given region, as the tumor tissue has been shown to be denser, and therefore brighter on a mammographic image.^7^^,^^21^^,^^22^ Correlations across the field in edge frequency and Fourier-based features, as seen in the mammogram radiomic features, has not been well documented in the literature and should be investigated further to understand how these relationships may relate to a potential field effect and breast cancer risk.

Kendall’s Tau-b correlation in radiomic features extracted from specimen radiographs showed slightly different results than those found in the mammograms. Similar to the mammogram results, the intensity-based and edge frequency features showed significant correlation with increasing distance from the tumor. The significant correlation in the intensity-based features in specimen radiographs could similarly be attributed to the underlying tissue density as represented in a brighter area in radiographic imaging, as the solid tumor mass tends to be denser, and therefore brighter.^7^^,^^21^^,^^22^ However, the significant correlations in GLCM features, as opposed to the Fourier-based features seen in the mammogram analysis, show a different aspect of the tissue structure exhibiting the correlation with increasing distance from the tumor. This change in the specimen radiographs could result from the tissue structure changing representation when removed from the body or a result of imaging with a different system. For deep learning features extracted from specimen radiographs, only the second feature or principal component showed a statistically significant correlation with ROI region location. It can be seen from Fig. 5 that the first feature indicated lower values on average for tumor ROIs than near or far ROIs but, the first feature did not show as linear a correlation with ROI region as feature 2 and failed to show statistical significance after multiple comparisons correction. Since these features do not have intuitive meanings in the same way that radiomic features do, the exact reasoning for this is not fully understood and may be investigated further in a future study. However, the first principal component for specimen radiographs may describe a characteristic that is not strongly correlated with ROI regions, as many radiomic feature categories are also not strongly correlated with ROI regions. While it is understood by the authors that evaluating specimen radiography is not typical clinical practice for risk assessment, investigating these features offers a unique opportunity to gain a more fundamental understanding of the field effect in both *in*- and *ex-vivo* imaging. Further, to the knowledge of the authors, this is the first study to evaluate radiomic features of mastectomy specimens.

Pearson correlation analysis between mammogram and specimen radiographs features for both radiomic and deep learning extended the results to indicate statistically significant correlations in both feature types across *in*- and *ex-vivo* imaging. For radiomic features, this was shown primarily in the intensity-based histogram features, indicating that features describing tissue intensity were highly correlated between mammograms and specimen radiographs. Structure based features failed to show these statistically significant correlations in radiomic features, which may be explained by changes in the tissue presentation when removed from the body or changes resulting from using a different imaging system. For deep learning features, only the first feature reached statistical significance. Given that the first feature is the first principal component, it describes the largest percentage of variance of all deep learning features, indicating a correlation between fundamental characteristics of the mammographic and specimen radiograph deep learning features.

Previous work by Baughan et al. investigated similar characteristics of the breast parenchyma in mammograms using radiomic features.^23^ While the results of the prior work indicated similarity of parenchymal signatures across the entire breast, it is important to note that the authors used a different statistical test (Kolmogorov Smirnov) and had also aligned the means of each distribution for each comparison. Such alignment of the distribution means removed the impact of the absolute feature value change and only investigated similarity of distribution shape. Not aligning the distribution means particularly influences histogram or intensity-based features and power law beta features, since their absolute values correlate to average gray values in the ROI. Thus, those prior summary results agree with the results presented in our current extended study.

Implications of these results and future studies may influence how patients are designated as high versus average risk of breast cancer. This will require future studies that better describe the physical extent of the cancer field for each tumor subtype and that quantify the risk associated with the mammographically derived cancer field.

It is important to note that statistical significance here is for the purposes of discovery only, not to indicate a clinical difference between two groups. For this reason, the Benjamini-Hochberg multiple comparisons correction was selected to be added to the statistical analysis. Other common multiple comparisons corrections, such as Bonferroni, control the family-wise error rate and may be overly conservative when the number of comparisons is large, and the potential cost of a false positive is relatively low. Identifying and emphasizing subgroups of features with a majority of significant correlations also helps to reduce the probability of a false positive conclusion and does not focus on single-feature results.

There are notable limitations to this study. First, it should be acknowledged that the results of this work have yet to be confirmed in a second independent cohort of women with cancer. Future work should confirm the same groups of features show statistically significant correlations with ROI location in a new cohort to improve robustness. This work also does not directly quantify the robustness of the ROI locations selected, and how that may impact feature values. While similar methods have been investigated for classification tasks, ROI location robustness should also be investigated in the future for describing the cancer field.^21^ One primary limitation of this work was the focus only on the features of women with breast cancer. However, this work aimed to characterize features of women with breast cancer in order to gain an understanding of potential signatures of a mammography-based field effect. Future work will look to incorporate these findings into classification models for malignant versus low risk and malignant versus benign lesions. Since this analysis only investigated features from images of women with confirmed breast cancer, density values were not controlled for, and the results likely represent an average distribution of breast densities; however, this will be investigated in future studies. The results also did not stratify findings by the molecular subtype of breast cancer present for each woman, due to the low number of patients within each subtype. The dataset of 74 total patients included 26 hormone receptor-positive/HER-2 negative tumors, 25 HER-2 positive tumors, and 23 triple-negative tumors. However, future analysis may find that feature relationships or presentation of a field effect may be more prevalent for certain molecular subtypes, just as the clinical profile and treatment of each molecular subtype varies.

## 5 Conclusions

The results of this study identified several characteristics of a potential mammography-based cancer field effect using human-engineered radiomic and deep leaning-based features from women with breast cancer. Radiomic analysis within mammograms indicated that features in the subcategories of intensity-based and Fourier-based features from ROIs closer to the tumor tended to show more similarity to the tumor than features from distant ROIs. Radiomic analysis in corresponding images of specimen radiographs showed similar results in intensity-based and GLCM features. Integration of novel data from specimen radiograph radiomic features showed statistically significant relationships of intensity-based features across the parenchymal field in *in*- and *ex-vivo* imaging. In deep learning features, similar relationships were found in both mammograms and specimen radiographs within the first two principal components. These results provide potential support for the presence of a cancer field effect, which is detectable from imaging studies alone, and support the development of computerized analysis of mammographic parenchymal patterns to assess breast cancer risk.

## 6 Appendix

Radiomic texture analysis features describing tissue contrast/intensity and structure in each breast region were calculated using an in-house workstation. Full feature descriptions and formulas can be found in the literature.^9^^–^^13^ Appendix Table 1 gives the category, name, and brief description of all 45 radiomic features calculated. These features are based on (a) fractal analysis, including box-counting and Minkowski methods; (b) edge-frequency analysis; (c) gray-level histogram analysis; (d) Fourier transform analysis; (e) the neighborhood gray-tone difference matrix; (f) Powerlaw beta from power spectral analysis; and (g) the gray-level co-occurrence matrix (GLCM).

**Table 1:** Categories, names, and brief descriptions of 45 radiomic features calculated for each ROI.

Category	Feature name	Description
(a) Fractal analysis, including box-counting and Minkowski methods	Boxcounting dimension	Fractal dimension estimated based on box-counting method
	Boxcounting dimension 1	Fractal dimension estimated based on box-counting method
	Boxcounting dimension 2	Fractal dimension estimated based on box-counting method
	Boxcounting dimension 3	Fractal dimension estimated based on box-counting method
	Boxcounting dimension 4	Fractal dimension estimated based on box-counting method
	Boxcounting dimension 5	Fractal dimension estimated based on box-counting method
	Minkod global MD	Fractal dimension estimated based on Minkowski method
(b) Edge-frequency analysis	Edge frequency: mean gradient	Average of edge gradient
	Edge frequency: max gradient	Maximum of edge gradient
	Edge frequency: minimum gradient	Minimum of edge gradient
	Edge frequency: standard deviation gradient	Standard deviation of edge gradient
(c) Gray-level histogram analysis	Histogram average	Average gray value within ROI
	Histogram maximum CDF	Gray-level threshold yielding 95% of the area under the histogram of the region
	Histogram minimum CDF	Gray-level threshold yielding 5% of the area under the histogram of the region
	Histogram balance	Ratio of (95% threshold-average) to (average-5% threshold)
	Histogram seventy CDF	Gray-level threshold yielding 70% of the area under the histogram of the region
	Histogram thirty CDF	Gray-level threshold yielding 30% of the area under the histogram of the region
	Histogram quasi balance	Ratio of (70% threshold-average) to (average 30% threshold)
	Histogram skewness	Denseness measure used to characterize local tissue composition
(d) Features based on Fourier transform analysis	Fourier root mean square (FRMS)	Root-mean-square variation based on Fourier transform analysis
	Fourier first moment of power spectrum (FFMP)	First moment of power spectrum based on Fourier transform analysis
(e) Neighborhood gray-tone difference matrix	Coarseness	Coarseness measure calculated from neighborhood gray-tone difference matrix
	contrast	Contrast measure calculated from neighborhood gray-tone difference matrix
(f) Powerlaw beta from power spectral analysis	Powerlaw beta 1	Exponent beta estimated based on powerlaw spectrum analysis
	Powerlaw beta 2	Exponent beta estimated based on powerlaw spectrum analysis
	Powerlaw beta 3	Exponent beta estimated based on powerlaw spectrum analysis
	Powerlaw beta 4	Exponent beta estimated based on powerlaw spectrum analysis
	Powerlaw beta 5	Exponent beta estimated based on powerlaw spectrum analysis
	Powerlaw beta 6	Exponent beta estimated based on powerlaw spectrum analysis
	Powerlaw beta 7	Exponent beta estimated based on powerlaw spectrum analysis
	Powerlaw beta 8	Exponent beta estimated based on powerlaw spectrum analysis
(g) GLCM	GLCM contrast	Measure of local image variations
	GLCM correlation	Measure of image linearity
	GLCM difference entropy	Measure of the randomness of the difference of neighboring pixels’ gray-levels
	GLCM difference variance	Measure of variations of difference of gray-levels between pixel-pairs
	GLCM energy	Measure of image homogeneity
	GLCM entropy	Measure of the randomness of the gray-levels
	GLCM homogeneity	Measure of the image homogeneity
	GLCM information measure of correlation 1 (IMC1)	Measure of nonlinear gray-level dependence
	GLCM information measure of correlation 2 (IMC2)	Measure of nonlinear gray-level dependence
	GLCM maximum correlation coefficient	Measure of nonlinear gray-level dependence
	GLCM sum average	Measure of the overall image brightness
	GLCM sum entropy	Measure of the randomness of the sum of gray-levels of neighboring pixels
	GLCM sum variance	Measure of the spread in the sum of the gray-levels of pixel-pairs distribution
	GLCM variance	Measure of the spread in the gray-level distribution

## Data and Code Availability Statement

The data used for this manuscript, including mammogram and specimen images, ROIs, and calculated features, are not publicly available due to patient privacy and data sharing agreements.
